# Association of Male Circumcision with Women’s Knowledge of its Biomedical Effects and With Their Sexual Satisfaction and Function: A Systematic Review

**DOI:** 10.1007/s10461-018-2313-0

**Published:** 2018-10-24

**Authors:** Jonathan M. Grund, Tyler S. Bryant, Carlos Toledo, Inimfon Jackson, Kelly Curran, Sheng Zhou, Jorge Martin del Campo, Ling Yang, Apollo Kivumbi, Peizi Li, Naomi Bock, Joanna Taliano, Stephanie M. Davis

**Affiliations:** 1 Division of Global HIV and TB, US Centers for Disease Control and Prevention, Pretoria, South Africa; 2 Johns Hopkins Bloomberg School of Public Health, Baltimore, MD USA; 3 Division of Global HIV and TB, HIV Prevention Branch, US Centers for Disease Control and Prevention, 1600 Clifton Road, MS-E04, Atlanta, GA 30329 USA; 4 Jhpiego, Baltimore, MD USA; 5 Division of Public Health Information Dissemination (DPHID), Library Science Branch, Center for Surveillance, Epidemiology, and Laboratory Services (CSELS), Atlanta, GA USA

**Keywords:** Male circumcision, Women, Knowledge, Sexual satisfaction, Sexual function

## Abstract

Male circumcision (MC) is a key HIV prevention intervention for men in countries with high HIV prevalence. Women’s understanding of MC is important but poorly understood. We conducted a systematic review including women’s knowledge of MC’s biomedical impacts and its association with female sexual satisfaction and function through October 2017. Thirty-eight articles were identified: thirty-two with knowledge outcomes, seven with sexual satisfaction, and four with sexual function (N = 38). Respondent proportions aware MC protects men from HIV were 9.84–91.8% (median 60.0%). Proportions aware MC protects men from STIs were 14.3–100% (72.6%). Proportions aware MC partially protects men from HIV were 37.5–82% (50.7%). Proportions aware MC is not proven to protect women from infection by an HIV-positive partner were 90.0–96.8% (93.0%). No increases over time were noted. Women’s MC knowledge is variable. Education could help women support MC and make better-informed sexual decisions.

**Electronic supplementary material:**

The online version of this article (10.1007/s10461-018-2313-0) contains supplementary material, which is available to authorized users.

## Introduction

Three randomized controlled trials (RCTs) in sub-Saharan Africa have established that male circumcision (MC) provides men with partial protection from acquisition of HIV and some sexually transmitted infections (STIs) through heterosexual sex [1–3]. Based on these findings, the World Health Organization (WHO) and Joint United Nations Program on HIV/AIDS (UNAIDS) have recommended MC for HIV prevention in countries with generalized HIV epidemics and low MC coverage [4]. MC programs for HIV prevention have been launched in 14 “priority countries” in East and South Africa: Botswana, Ethiopia, Kenya, Lesotho, Malawi, Mozambique, Namibia, Rwanda, South Africa, Swaziland, Tanzania, Uganda, Zambia, and Zimbabwe [4]. Since 2009, MC has become a pillar of the global response to HIV in sub-Saharan Africa, as over 15 million circumcisions for HIV prevention have been conducted through 2016 [5].

While males benefit directly and individually from circumcision, increasing MC prevalence also has important implications for women. Substantial evidence supports indirect protection for female partners from HIV—believed to be due to indirect protection via decreased prevalence in men and thus a decreased risk of exposure to an infected male partner– and some STIs [4, 6–8]. The inverse, direct protection, would refer to decreased infectiousness of infected male partners resulting from circumcision, and is not proven to exist for HIV or STIs. WHO/UNAIDS’s recommendation for MC for HIV prevention explicitly mentioned that information needed to be communicated with women and communities about the extent of MC’s protective benefits, and MC programming is an opportunity to promote shared sexual decision-making, gender equality, and improved health for men and women [4].

While these biomedical impacts could improve women’s health outcomes in countries implementing MC, women need to have correct knowledge about the protective benefits and limitations of MC for themselves and their male partners in order to make informed decisions for their own sexual health. Due to the fact that males have been the primary recipients of MC outreach and education, women may have highly variable understandings of MC’s impact on and effect on their health [9–11]. Following the three RCTs, women’s knowledge was expected to increase as national MC for HIV prevention programs were established; however, it is unclear whether this has happened.

Since VMMC programs were established in several sub-Saharan African countries, demand creation activities in VMMC programs to date have tended to focus on education and recruitment campaigns for adolescent and adult men [9, 12, 13]. However, some recent studies have also investigated women’s acceptance of male circumcision and their partner’s readiness to undergo the procedure, and willingness to help promote VMMC for their male partners and contacts [10, 14]. Broader public health programs that promote women’s health could also benefit from better understanding of women’s knowledge and educational needs around MC and its implications for women. In addition, clarification of any association between MC and women’s sexual satisfaction and function and MC is important for understanding the full implications of MC for women. In order for MC to be more widely accepted and scaled up, a more comprehensive understanding of women’s knowledge about the procedure, and its impact on their sexual satisfaction and function, is needed to inform messaging.

We previously conducted a systematic literature review of the impact of MC on biomedical outcomes among women. Biomedical outcomes have been reported separately [6]; here we present the results that characterize the association of MC with female sexual satisfaction and function, and women’s knowledge regarding MC and its protective effects.

## Methods

Detailed methods for our literature search are presented elsewhere [6]. Briefly, searches were conducted in Medline, and in multiple other published and grey literature databases and conference abstracts. Multiple forms of the terms “women” and “circumcision” and multiple measures of sexual satisfaction or overall sexual function were used. Included knowledge outcomes were those that addressed one of these key knowledge elements: 1) that MC provides protection for men against HIV and/or STIs; 2) that this protection is only partial; and 3) that MC has not been proven to protect women against acquiring HIV from an infected male sexual partner [15].

Publications were included if they were written in English, reported primary data, described a sampling method, compared partners of circumcised and uncircumcised men in the case of sexual satisfaction and function outcomes, reported an accepted outcome, and were not identified as a duplicate. Accepted outcomes included publications in which the knowledge element elicited by the question asked was unambiguous. This was explicitly identified as a concern in the case of the ‘MC not proven to protect women’ outcome, where data based on questions that did not distinguish between “indirect” and “direct” protection to women were excluded.

Additionally, data were excluded for ambiguous questions measuring partial protection like “Does MC fully protect men from HIV?” that made it impossible to determine whether a respondent believed protection was only partial or did not believe protection existed at all. Articles were not excluded due to the “type” of circumcision discussed: adult/adolescent versus early infant male circumcision (EIMC), which is presumed to confer the same HIV- and STI-related benefits once a circumcised male neonate reaches sexual debut.

For sexual satisfaction and function outcomes, because of the requirement for comparison between personal sexual experiences of partners of circumcised and uncircumcised men, survey data on women’s general opinions of the effect of MC on these outcomes were not included. Data were reported with the same significant digits as provided in the source material. Estimates provided as proportions were converted to percentages to present all results on the same scale.

Qualitative studies were included, but it was determined a priori they would be presented only for outcomes on which no quantitative data was found.

**Fig. 1:**
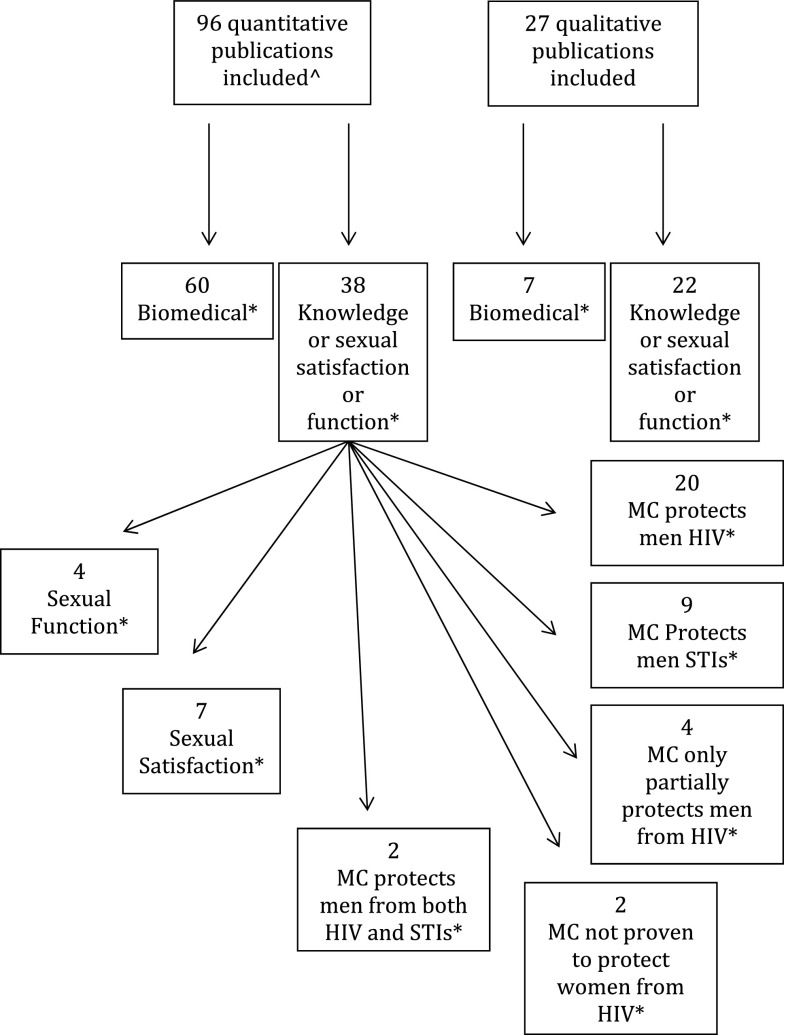
Article Flow Diagram. *Numbers do not sum to total in box above, as some publications provided both biomedical and knowledge data, more than one knowledge outcome, a biomedical outcome not included in this paper, and/or both qualitative and quantitative data. ^^^The number of articles included is larger than the number found in the original paper on biomedical outcomes. This is because a refresh search was performed for the knowledge, sexual satisfaction, and sexual function outcomes, and four new articles were found

**Fig. 2:**
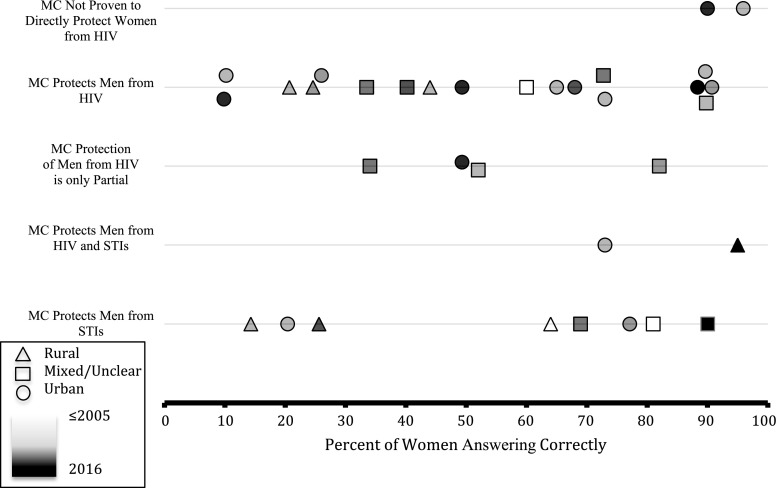
Percent of women with correct knowledge of male circumcision (MC) protection

**Fig. 3:**
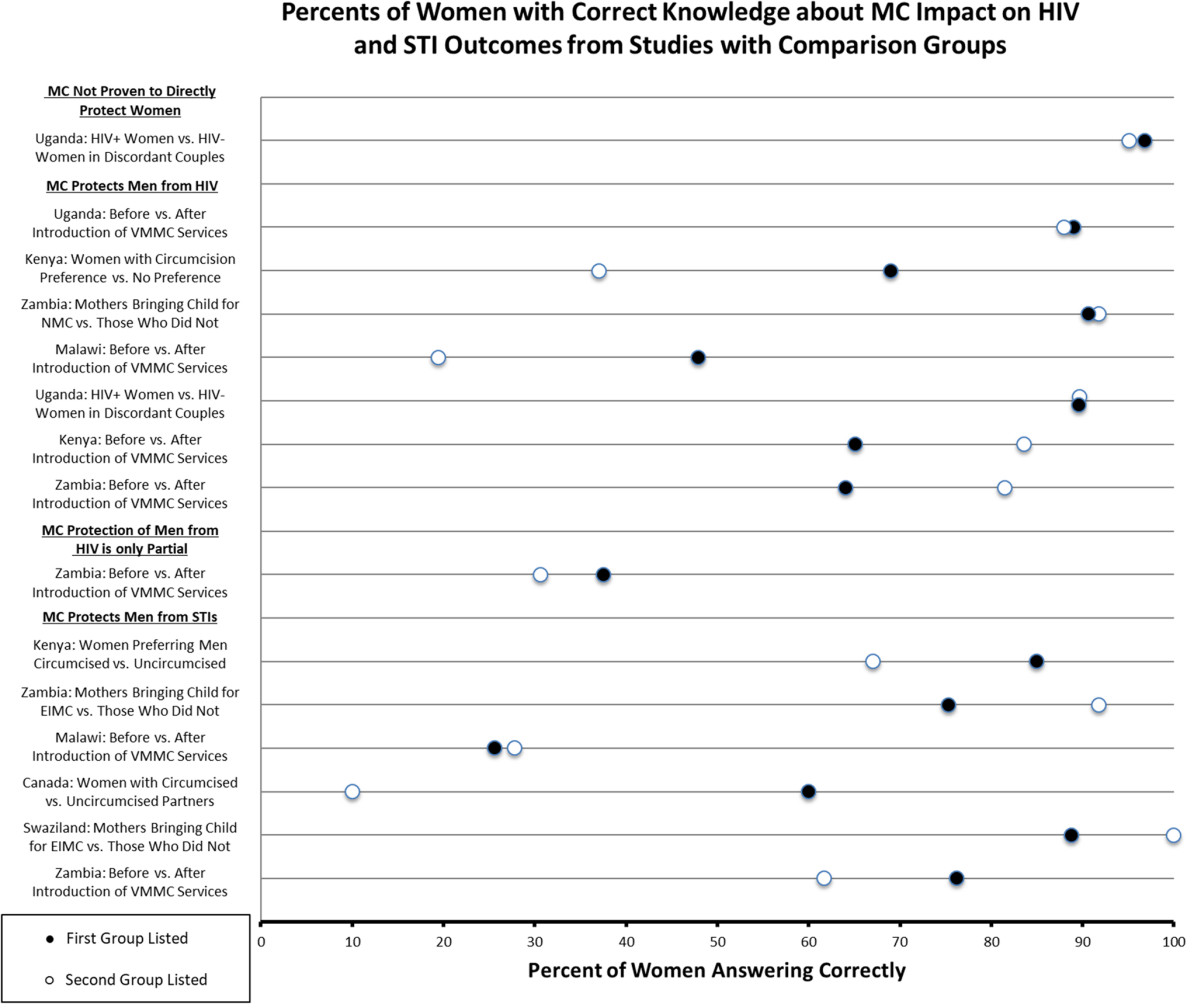
Percent of women with correct knowledge about MC impact on HIV and STI outcomes from studies with comparison groups

Publications were first title-abstract screened and then full-text screened, and abstracted by two trained researchers. Results were harmonized at each stage. Figure 1 shows the article inclusion pathway. Abstracted data included year of publication, study design, inclusion/exclusion criteria, diagnostic methods, sample sizes, and point estimates and uncertainty around impacts. See Supplementary Table for a full display of abstracted variables. Only the main result from each publication for each knowledge outcome is displayed in Fig. 2, the summary of results, to avoid inflating the apparent weight of publications reporting on multiple subgroups. Individual abstracted data points were excluded if they were identified as earlier duplicates of later included data points (e.g. derived from an abstract on data later published in manuscript form). An associated manuscript’s [6] online Supplement Appendix 2 includes detailed methods [16]. In cases when there is no point estimate given for the entire sample and for which a combined point estimate could not be calculated, these are excluded from Fig. 2 and are highlighted in Supplementary Table 1 with a red background. Figure 3 shows comparison between groups within studies that examined subgroups.

Quality grading for RCTs used the GRADE criteria [17] that ranks publications as providing high-, moderate-, low- or very low-quality evidence. Quality grading for observational studies used Newcastle–Ottawa or Newcastle–Ottawa-based scales [18]. These grading scales award points for the quality of evidence in a study depending on its comprehensiveness and justification. Maximum quality scores were adapted by removing the points assigned to quality elements graded in the Newcastle–Ottawa scales which were inconsistent with the purpose of the knowledge studies included. For cohort studies, the maximum score was set to 9 instead of 10, as self-report is an acceptable measure for a knowledge outcome. For cross-sectional studies comparing two groups, the maximum score was set to eight for the same reason. For cross-sectional studies, the original scales are designed for studies making comparisons between two groups, so quality scoring was modified for those knowledge studies that reported a single-group cross-sectional knowledge outcome, removing points for quality of between-group comparisons from the maximum potential score [18]. The final maximum score for these was set to 4 instead of 10, and for cross-sectional studies comparing two groups it was 9, as self-report was also acceptable here.

Quality of the overall body of evidence on each outcome was then assessed, using a modified Child Health Epidemiology Research Group (CHERG) criteria format [19] (Table 1). The CHERG guidelines reflect the quality of each individual publication’s data on that outcome and generalizability of results to the population and intervention of interest (women in the general population in countries with generalized HIV epidemics; and male circumcision, respectively). In the case of the sexual satisfaction and function studies, which compared exposed to unexposed groups, consistency of effect between publications was also included. This was determined for each outcome via a pre-specified algorithm incorporating study design and number and statistical significance of studies [6].

**Table 1:** Summary of publications reporting on the impact of MC on health outcomes in women

Outcome	Number of studies, publications	Design(s)	Region(s)	Median quality score among studies reporting a single combined point estimate*(RCT; observational)	Generalizability to area/population of interest**	Age range (in years)
MC not proven to protect against male-to-female transmission	2, 2	Cross-sectional	Africa	No RCT; 2/7	High	15+
MC protects men from HIV	16, 19	Cross-sectional, Cohort	Africa, North America	No RCT; 3/7	High	15+
MC protection against HIV is partial	3, 4	Cross-sectional, Cohort	Africa	No RCT; 2/7	High	15+
MC protects against both HIV and STIs	1, 1	Cross-sectional	Africa	No RCT; 2/7	High	18-35
MC protects men from STIs	9, 9	Cross-sectional, Cohort	Africa, North America	No RCT; 2/7–3/7	High	15+
Sexual satisfaction	6, 6	RCT, Cross-sectional	Africa, North America, Europe	Unclear; 2/8	High	15+
Sexual function	4, 4	Cross-sectional	Australia, North America, Europe	No RCT; 2/8–3/8	High	16+

*Excludes studies which only reported comparisons between two groups

**Based on the modified CHERG definition of “generalizability”

## Results

The primary article exclusion flowchart has been presented elsewhere [6]: Fig. 1 shows the detailed exclusion flowchart for knowledge, sexual satisfaction, and sexual function outcomes. A total of 38 articles were identified with 32 having data regarding women’s knowledge of MC, seven with data on sexual satisfaction, and four with data on sexual function. Among knowledge publications, 20 presented data on women’s knowledge that MC protects men from HIV, nine on knowledge that MC protects men against STIs, and two on knowledge of both. Four articles reported on knowledge that MC only provides partial protection from HIV for men, and two reported knowledge that MC is not proven to protect against male-to-female HIV transmission. No articles were found with quantitative data examining knowledge that MC only partially protects men from STIs. Figure 2 includes all knowledge studies pre- and post-2007 for which a combined single point estimate was reported or could be calculated. Table 1 includes all studies, with median quality scores for those reporting a combined single point estimate. For each knowledge element, the following subsections summarize overall findings including range and median, any subgroup findings, and any findings involving comparisons between groups or across time periods.

Results are presented in two major categories: first women’s knowledge, and then women’s sexual satisfaction and function. Within the knowledge category, results are presented under a separate subheading for each pre-specified knowledge outcome. Knowledge outcomes with quantitative data first present the descriptive summary measures including median and range of overall knowledge and identify extremes; then present findings specific to any population subgroups analyzed. Knowledge outcomes with no quantitative data present a description of the findings from qualitative studies of the outcome. Satisfaction and function outcomes instead discuss the consistency and quality of evidence around these associations because these dealt with associations with circumcision and used heterogeneous measures not amenable to a descriptive summary.

### Women’s Knowledge

Most studies reporting quantitative data on women’s knowledge were located in countries in sub-Saharan Africa: Kenya, South Africa, Uganda, Zambia, Namibia, Botswana, Swaziland, Zimbabwe, and Malawi. However, data on women’s knowledge about protection for men from HIV and/or STIs were also available from Kingston, Jamaica; Ontario, Canada; and Papua New Guinea.

The majority of articles contained data from cross-sectional surveys with knowledge questions, some with comparisons between subgroups defined by HIV status, partner circumcision status, or other characteristics. Other articles presented data from serial cross-sectional or cohort studies following temporal trends, including before and after MC scale-up. The most common quality limitations were non-representativeness due to nonrandom sampling, small sample sizes, and heterogeneity of questions and question clarity between studies. Given these limitations, most studies of women’s knowledge were of low quality.

### Knowledge: MC Protects Men from HIV

Proportions of respondents with correct knowledge that circumcision reduces HIV risk for men ranged from 9.8 to 91.8%, with a median of 60.0%. The lowest proportions were found among partners of HIV-negative uncircumcised men recruited from a community clinic in Zambia (9.8%) [20], and women recruited from an STI clinic in Kingston, Jamaica (10.2%) [21]. Subgroups (groups clearly distinct in some key feature from the general population, whether they constituted the whole study population or a subgroup within it) that were examined among studies reporting this outcome included women with uncircumcised partners, women who were HIV-positive, women who brought or did not bring their newborns for EIMC, and HIV-positive and -negative women in HIV-serodiscordant relationships. Among these, all had scores that were near the general median or higher, except for the group of women with uncircumcised partners who did not have circumcision preferences, who had 36.7% correct knowledge [22].

Among studies comparing different subgroups on knowledge of protection against HIV for men, a study in Zambia comparing women who brought their newborns in for EIMC (98.1% correct) to those who didn’t (90.6% correct), found a non-statistically significant odds ratio of 1.01 [23]. A study in Uganda of HIV serodiscordant couples comparing by female partner serostatus also showed no statistically significant difference between the two groups, with 89.7% correct among HIV-positive women and 89.6% correct among HIV-negative women [24]. Another study in Uganda reported that 88.6% of women who supported MC believed that it prevents HIV, while only 11.4% of those not supporting MC reported correct knowledge [25]. All studies comparing knowledge before and after an intervention—either a MC program launch or a circumcision research intervention—showed an improvement in the proportion of women correctly answering that circumcised men were protected from HIV (Fig. 3). The largest improvement was 28.5% from a study conducted as part of an intervention named CARE-Malawi (19.4–47.9%; no *p* value reported) [26], with the others having statistically significant absolute increases of 18.5% (65.1–83.6%, p < 0.001) and 17.5% (64.0–81.5%; p < 0.001) in Kenya and Zambia, respectively [27, 28].

### Knowledge: MC Protects Men from STIs

The nine publications reporting on women’s knowledge that MC protects men from STIs had percentages of correct answers ranging from 14.3 to 100%, with a median of 72.6%. The publication with the lowest percentage of correct knowledge for this outcome was the study conducted in Malawi, among the wives of participants in a circumcision, information, and HIV prevention project [29]. Subgroups considered for this outcome include women with uncircumcised partners and women who brought or did not bring their newborns for EIMC. Compared to the median percent correct of all studies for this outcome, every subgroup performed better.

Five of these publications compared outcome data between two groups of women. In a study comparing women who chose to have their children circumcised to those who did not, 100% of women with circumcised children answered correctly that male circumcision protects men from STIs, while 88.8% of women with uncircumcised children answered correctly [30]. The difference between the two groups was not statistically significant. However, there was a statistically significant difference between women who brought their children for EIMC (91.8% correct) and those who did not (75.3% correct) in another study (OR: 3.39; 95% CI: 1.51–7.61; p = 0.003) conducted in Zambia [23]. There was a statistically significant increase of 14.5% (61.7–76.2%; p = 0.001) in knowledge for this outcome after the MC program scale-up in Zambia [28]. However, in the CARE-Malawi intervention study, knowledge that MC protects men from STIs was stable over time (27.8% pre-intervention, 25.6% post) [26].

### Knowledge: MC Protects Men from HIV and STIs

There were two publications with a composite outcome on knowledge that MC provides protection from both HIV and STIs. In a qualitative in-depth interview of 30 women in Kisumu, Kenya, 73% answered correctly [31]. The other study, including 613 women in Homa Bay County, Kenya, found that 95% of women answered correctly [32].

### Knowledge: Protection Conferred to Men by MC Against HIV and STIs is Partial

The proportion of individuals with correct knowledge that MC only partially protects men from HIV in the four studies that passed screening ranged from 37.5 to 82%, with a median of 50.7%. The included subgroup, postpartum mothers, had essentially the same percentage of correct knowledge, at 51.7%, than the overall median [33]. The study making a comparison of women’s knowledge before and after a MC program scale-up found a statistically significant increase of 6.9% in correct answers (30.6–37.5%; p < 0.001) [28].

The search returned no publications with quantitative data that addressed knowledge that MC only partially protects men from STIs. Qualitative results on this outcome are discussed below.

### Knowledge: MC is Not Proven to Protect Women from Acquiring HIV from Infected Partners

In the two studies providing data for this knowledge outcome, the percentage of women surveyed who had knowledge of MC not being proven to protect women from acquiring HIV from an HIV-positive partner ranged from 90.0 to 96.8%, with a median of 93.0%. Studies were conducted in South Africa and Uganda. Among a subgroup of women in serodiscordant relationships, the Ugandan study compared the knowledge of HIV-positive women (96.8%) to that of HIV-negative women (95.1%); the difference was not significant [24]. This result was similar to that found in a non-included study surveying the general population of South Africa with 90.0% correct knowledge [34].

### Qualitative Data: Women’s Knowledge: Protective Effect of MC Against HIV is Partial

A total of 22 articles presented only qualitative data on knowledge, sexual satisfaction, and sexual function outcomes using in-depth interviewing and focus group discussions. Only one outcome, women’s knowledge that the protective effect of MC against HIV for men is only partial, lacked any articles with quantitative data, and therefore the results of the ten qualitative articles addressing this outcome are briefly discussed here. This lack of quantitative data is primarily because questions relevant to this outcome were ambiguous with respect to whether the questions investigated women’s knowledge of any or of partial protection of MC.

The ten qualitative articles primarily reported parents’ knowledge of EIMC and women’s knowledge of MC protection and risk. In the two articles exclusively addressing knowledge of EIMC, in Zambia and Swaziland [35, 36], mothers of infant sons generally had accurate knowledge about the benefits and risks of male circumcision surgery and expressed the intention of circumcising their infant sons, though the preferred age of circumcision varied from infancy to adolescence. The eight remaining articles contained qualitative data from several countries in sub-Saharan Africa and the United States. Women’s knowledge of MC’s partial protection against HIV varied considerably, and some overestimated the protection MC conferred against some STIs [28, 31, 37–42].

### Women’s Sexual Satisfaction

There was high-consistency evidence for a positive association between MC and women’s sexual satisfaction, based on seven included publications. Measures were heterogeneous across publications, including level of sexual satisfaction [43, 44], incomplete sexual needs fulfillment [45], and overall rating of the sexual experience [47]. No RCTs compared satisfaction between circumcision intervention arms, but three surveys were included of female partners of men who had received MC for HIV prevention, comparing their sexual satisfaction before and after the men’s procedures. These included partners of participants in the Ugandan circumcision RCT, of whom 97.1% reported improvement or no change and 2.9% with decreased satisfaction [41]; partners of participants in the Zambian Spear and Shield project [47] (62.2% improvement, 15.7% no change, 13.2% decreased, 8.8% no opinion); and partners of Kenyan men followed longitudinally (91% reported improvement, other responses not presented) [48], all supporting a beneficial impact of MC. In addition, one cross-sectional study of women in South Africa reported nonsignificant increases in women’s perceived pleasure during sex with a circumcised partner, though it was not clear if these women had actual sexual experiences with circumcised men, or if these were assumed perceptions [44].

One additional publication that was excluded from the main review due to being published in Spanish used a pre-post design in Mexico and showed nonsignificant improvements in sexual satisfaction after circumcision [49].

However, female respondents to cross-sectional surveys in other settings—Canada [50], Denmark [45], and an unspecified location [46]—reported significantly higher satisfaction with uncircumcised partners. Two of these studies recruited volunteers using advertisements, one including placement in an anti-circumcision newspaper [46], though respondents in the other conversely expressed a preference for circumcised partners [50]. The third was a nationally representative survey in Denmark [45]. Prevalence of male circumcision in the Danish survey was 3.7%, reflecting the rarity of circumcision in Denmark.

### Women’s Sexual Function

In contrast, for sexual function, there was indeterminate consistency evidence, because only the Danish survey presented data on this measure, reporting an adjusted odds ratio of 3.26 (95% CI 1.15–9.27; p-value not reported) for answering “often” or “every time” to whether they had any sexual function difficulties among women with circumcised as compared to uncircumcised partners [43]. However, all three cross-sectional publications presented data on individual components of sexual function, which were not prespecified outcomes: orgasm ease, arousal, pain, and lubrication. The Canadian publication [50] reported nonsignificant and minimal differences for orgasm ease, arousal, pain, and lubrication, with slightly better outcomes for circumcised partners for the first two and for uncircumcised partners for the second two. The studies from Denmark and an unspecified location did not examine arousal but reported improved outcomes with uncircumcised partners with the other metrics. In a fourth study of women in Australia recruited through unspecified community groups [51], partner circumcision was associated with vaginal dryness (see Supplemental Table 1 for details).

For both satisfaction and function outcomes, publications reporting evidence in both directions were of low or medium quality, with scores ranging from 1 to 5 out of a maximum quality score of 8, except for the Rakai RCT follow-up.

## Discussion

### Women’s Knowledge

The evidence collected in this review highlights the large variability in key knowledge areas regarding MC globally, but particularly across sub-Saharan Africa. Correct knowledge about MC is important for women’s reproductive health decisions. HIV-negative women who understand that a partner’s circumcision does not remove the risk of HIV transmission can protect themselves; HIV-positive women who understand that they can still transmit the virus to circumcised men can protect their partners; and all women can provide informed input to decisions around circumcision for partners and male family members [31]. From a public health context, this support can enhance the uptake of MC [14].

Subgroups examined consisted of women who brought or did not bring their sons for EIMC, women who were HIV-positive, women in HIV-serodiscordant relationships, and women with uncircumcised partners. Each of these subgroups consistently performed near or better than median of all knowledge scores across all outcomes, which provides some reassurance about knowledge levels of women in some of the groups for which the impact of MC is most important.

The overall quality of included studies was low. However, the study with the highest quality score (8/9) showed that enrolled women in Uganda were knowledgeable (> 89% answering correctly) that circumcision protects men from HIV but does not necessarily protect women from HIV if their partners are HIV-positive.

Methods for collecting data varied among studies, including face-to-face structured and in-depth interviews or surveys with differently worded questions, lengths, and structures. Studies from Jamaica, Malawi, and Zimbabwe reported the lowest knowledge, but there was not a clear regional difference in knowledge levels between southern and eastern Africa overall, and knowledge levels were generally higher in African vs. non-African settings.

Although the majority of individual studies making pre-post comparisons in specific settings where MC was scaled up did show higher level of women’s knowledge at the later time point (Fig. 3), basic knowledge remained far from universal, and there was no discernable pattern of increasing knowledge over time in the overall dataset (Fig. 2). Even one publication that compared women’s knowledge from 2007-2012 in a specific setting that experienced rapid MC scale-up found no significant increase [44]. This observation could have resulted from MC educational efforts being primarily directed at men. More generally, while it seems intuitive that MC scale-up would bring some knowledge benefits for women, the specific areas studied over multiple time points may have been impacted by the studies themselves and overrepresented this impact. The highest-impact educational intervention from CARE-Malawi resulted in a 28.5% increase in women answering that MC protects men from HIV. However, this same study found a decreased percentage of women answering correctly that circumcised men were protected from STIs after the intervention. The reason for this is not clear; it could be a result of the promotion of MC programs primarily as a tool for HIV prevention. Women in other communities in sub-Saharan Africa receiving MC services may benefit from similar educational interventions, although these types of intervention should be evaluated to determine if women-directed campaigns could successfully increase women’s knowledge, and if so, how they could be rapidly introduced in conjunction with VMMC programs. In general, knowledge also appeared higher in urban than in rural communities (Fig. 2).

Limitations included the unavoidable exclusion of substantial data where it was ambiguous what knowledge elements were tested, and judgments on the clarity of article questions, and thus eligibility for inclusion, were inherently subjective. Additional limitations involve the low quality of most included studies, particularly due to risk of non-representativeness of the general population due to sampling methods used. Studies that were published after our October 2017 cutoff are not included in our analysis. One study from 2018 on South African women in a high burden area of Cape Town found that approximately two-thirds of women were unaware that male circumcision partially protects men from HIV [11]. However these results were in line with our findings and would not have substantially altered our results. It does highlight the continued need to address women’s knowledge of MC’s benefits.

Future studies of women’s knowledge can benefit from ensuring that questions unambiguously elicit the knowledge element of interest, about the group of interest, in this complex area: e.g., “If a man with HIV gets circumcised, does this make him less likely to give HIV to a woman?” more clearly targets the direct effect than “Does circumcision for men protect women from getting HIV?”. Similarly, “Does getting circumcised decrease a man’s risk of getting HIV?” more clearly identifies the group (men) being considered than “Is preventing HIV a benefit of circumcision for men?”. While assessing the precision of survey questions was not an original objective of this review, it is an important finding.

### Sexual Satisfaction and Function

Evidence around impact on sexual satisfaction and function is complex and inconclusive. Sexual satisfaction has a substantial cognitive and emotional component that may be highly influenced by beliefs about the impact of circumcision on risk of HIV and STIs, or by traditional or personal preferences regarding circumcision status. In addition, it may be expected to vary naturally for individuals over time for reasons unrelated to interval changes in circumcision status. The striking difference between sexual satisfaction findings in sub-Saharan Africa and elsewhere emphasizes the likely contribution of cultural norms.

Most studies of sexual satisfaction and function had potential for selection bias and an incomplete description of the measurement tools used. This led to low quality ratings and made interpreting the validity of these results particularly challenging. There were several concerns with the populations and methods used in the studies. For instance, given the low circumcision prevalence in the Denmark sample, many of participants’ male partners may have been circumcised for medical reasons, though the potential impact of this on participants’ satisfaction is unclear. The non-representative sampling methods used in the other included studies could have led to inclusion bias both inside and outside sub-Saharan Africa, potentially in opposing directions. Additionally, given the heterogeneous measures of sexual satisfaction included between studies, it is uncertain whether all of these captured the same complex outcome. Despite these limitations, we consider the African studies more applicable and valid than the others to sexual satisfaction among female partners of MC clients, by virtue of both their setting and their use of each man as his own control. The same confounders apply to the measures of individual components of sexual function, but the African pre-post publications did not examine these metrics, so the data is even more limited.

Therefore, the overall evidence base is reassuring that male circumcision in sub-Saharan Africa does not decrease sexual satisfaction among female partners, but evidence collected elsewhere suggests it may be associated with changes for the better and worse in specific elements of their sexual function. Gaps remain in our understanding of influencing and confounding factors that explain variations in findings between settings, the importance of specific components of sexual function to satisfaction, and the extent to which current metrics capture these elements (e.g., vaginal lubrication is not plausibly directly affected by partner circumcision status, but could be mediated by arousal, or may actually convey differences in friction.) The relationship between male circumcision, specific components of sexual function in women, and sexual satisfaction deserves further investigation in pre-post studies, ideally where reasons for obtaining circumcision are unrelated to sexual function.

## Conclusions

Women’s knowledge of the effects of male circumcision is highly variable both within and across countries and is not clearly improving over time. Addressing this as an educational part of MC program scale-up could allow women to play a more active role in promoting male circumcision in their communities and make better-informed decisions around their personal sexual health. In order to reach current ambitious global HIV prevention and treatment goals [52], HIV prevention programs must prioritize high-impact interventions to the individuals most at risk. Women’s knowledge about male circumcision is critical, especially as VMMC programs focus on appealing to high-risk men. The overall impact of male circumcision on women’s sexual satisfaction is benign, but specific impacts on elements of sexual function are not well understood and could benefit from further investigation by representative, high-quality studies.

## Electronic supplementary material

Below is the link to the electronic supplementary material.

Supplementary material 1 (XLSX 78 kb)
